# MicroRNA-146a Deficiency Protects against *Listeria monocytogenes* Infection by Modulating the Gut Microbiota

**DOI:** 10.3390/ijms19040993

**Published:** 2018-03-26

**Authors:** Chong-Tao Du, Wei Gao, Ke Ma, Shui-Xing Yu, Na Li, Shi-Qing Yan, Feng-Hua Zhou, Zhen-Zhen Liu, Wei Chen, Lian-Cheng Lei, Yong-Jun Yang, Wen-Yu Han

**Affiliations:** 1 Key Laboratory of Zoonosis, Ministry of Education, College of Veterinary Medicine, Jilin University, Changchun 130062, China; duct@jlu.edu.cn (C.-T.D.); make17@mails.jlu.edu.cn (K.M.); yushuixing@hotmail.com (S.-X.Y.); lna17@mails.jlu.edu.cn (N.L.); 65166666@163.com (S.-Q.Y.); zhoufh15@mails.jlu.edu.cn (F.-H.Z.); 18243189542@163.com (Z.-Z.L.); chw_cc@jlu.edu.cn (W.C.); leilc@jlu.edu.cn (L.-C.L.); 2 College of Animal Sciences, Jilin University, Changchun 130062, China; gaowei2004jlu@126.com; 3 Jiangsu Co-Innovation Center for the Prevention and Control of Important Animal Infectious Disease and Zoonoses, Yangzhou University, Yangzhou 225009, China

**Keywords:** microrna-146a, gut microbiota, *Listeria monocytogenes*, bacterial infection

## Abstract

The gut microbiota and microRNAs play important roles in the defense against infection. However, the role of miR-146a in *L. monocytogenes* infection and gut microbiota remains unclear. We tried to determine whether miR-146a controlled *L. monocytogenes* infection by regulating the gut microbiota. Wild-type and miR-146a-deficient mice or macrophages were used to characterize the impact of miR-146a on animal survival, cell death, bacterial clearance, and gut microbiota following *L. monocytogenes* challenge. We found that *L. monocytogenes* infection induced miR-146a expression both in vitro and in vivo. When compared to wild-type mice, miR-146a-deficient mice were more resistant to *L. monocytogenes* infection. MiR-146a deficiency in macrophages resulted in reduced invasion and intracellular survival of *L. monocytogenes*. High-throughput sequencing of 16S rRNA revealed that the gut microbiota composition differed between miR-146a-deficient and wild-type mice. Relative to wild-type mice, miR-146a-deficient mice had decreased levels of the *Proteobacteria* phylum, *Prevotellaceae* family, and *Parasutterella* genus, and significantly increased short-chain fatty acid producing bacteria, including the genera *Alistipes*, *Blautia*, *Coprococcus_1,* and *Ruminococcus_1*. Wild-type mice co-housed with miR-146a-deficient mice had increased resistance to *L. monocytogenes*, indicating that miR-146a deficiency guides the gut microbiota to alleviate infection. Together, these results suggest that miR-146a deficiency protects against *L. monocytogenes* infection by regulating the gut microbiota.

## 1. Introduction

*Listeria monocytogenes* (*L. monocytogenes*) is the causative agent of the systemic infections called listeriosis [1]. These are a foodborne infections caused by food contamination by bacteria [2]. Listeriosis affects newborns, pregnant women, older adults, and adults with weakened immune systems [3]. *L. monocytogenes* can cross the intestinal, blood-brain, and feto-placental barriers, leading to gastroenteritis, meningoencephalitis, and maternofetal infections [4]. *L. monocytogenes* has been used as a model organism to study host-pathogen interactions [5].

MicroRNAs (miRNAs) are endogenous, small non-coding RNAs that regulate most important cellular processes by regulating target gene expression at the post-transcriptional level by targeting mRNAs for translational repression or degradation [6]. MiRNAs have emerged as key regulators of host immune responses [7]. Recent studies have shown that miRNAs play important roles in the defense against bacterial infections [8,9,10]. Regulation of miRNA expression has become increasingly recognized as a novel molecular strategy exploited by intracellular bacterial pathogens to manipulate host cellular pathways to survive in host cells [11]. Previous studies have revealed that microRNA-146a (miR-146a) is involved in inflammation and bacterial infections. MiR-146a negative regulates LPS-activated TLR and NFκB signaling pathway by targeting TRAF6 and IRAK1 [12,13]. Following *Helicobacter pylori* infection, the increased miR-146a inhibits bacteria-induced inflammation by targeting IRAK1 and TRAF6 [14]. MiR-146a also promotes mycobacterial survival by repressing NO production via targeting TRAF6 [15]. These data suggest that miR-146a plays an important role in health and disease [16], but it remains elusive whether miR-146a can regulate infection with *L. monocytogenes*.

The gut microbiota has a major impact on a range of metabolism, nutrition, nervous, endocrine, and immune functions [17,18,19,20,21]. It plays a critical protective role in host defense against infection, both by mediating colonization resistance and by modulating potent innate immune responses [18]. Changes in the gut microbiota can promote or confer resistance to bacterial infection. The gut microbiota is regulated by multiple factors, including diet, disease states, host genetics, age, and environmental factors [22]. Since miRNAs play important roles in defense against bacterial infections, it is important to understand the interaction between gut bacteria and miRNAs. However, it remains unclear whether miRNA can regulate the gut microbiota.

In this study, we describe our new discovery about the regulatory role of miR-146a during *L. monocytogenes* infection. We aimed to determine whether miR-146a controls *L. monocytogenes* infection by regulating the gut microbiota.

## 2. Results

### 2.1. MiR-146a Expression Is Induced by L. monocytogenes Infection both In Vitro and In Vivo

**Figure 1:**
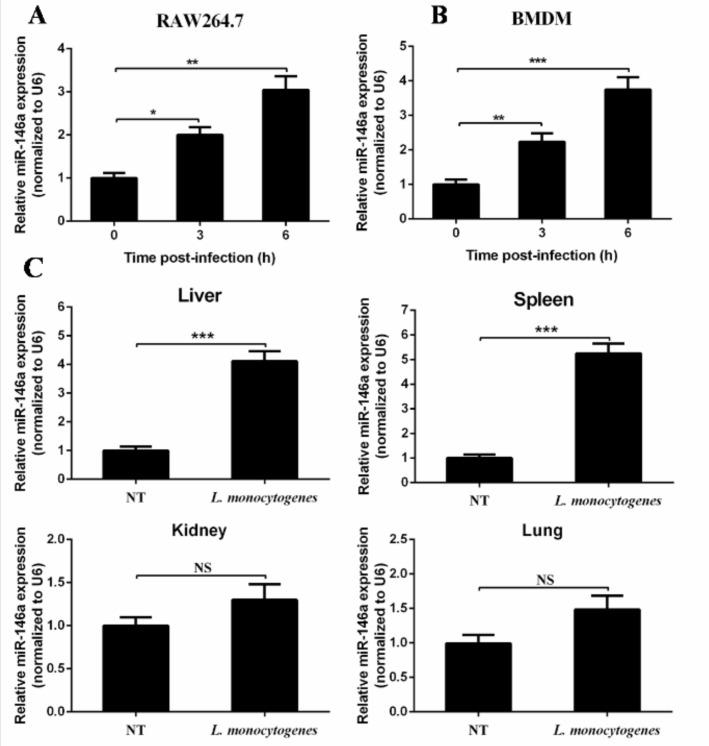
*L. monocytogenes* infection induced miR-146a expression both in vitro and in vivo. RAW264.7 cells (**A**) or Murine bone marrow-derive macrophages (BMDMs) (**B**) were infected with *L. monocytogenes* 10403S at a multiplicity of infection (MOI) of 20 for the indicated time. Different organs were collected from *L. monocytogenes*-infected or PBS-treated mice at day 3 post-infection. MiR-146a was measured by real-time PCR, with a total of six mice per group (**C**). Data are shown as the mean  ± s.e.m. of three independent experiments. * *p*  <  0.05; ** *p*  <  0.01; *** *p*  <  0.001; NS, no significance.

To explore the expression of miR-146a during *L. monocytogenes* infection, the macrophage-like cell line RAW264.7 and murine primary bone marrow-derive macrophages (BMDMs) were challenged with *L. monocytogenes* 10403S. Real-time PCR was used to determine the expression level of miR-146a. The level of miR-146a in RAW264.7 cells at 6 h post-infection was approximately three-fold higher than that in the control (Figure 1A). Similarly, miR-146a expressions were elevated in BMDMs after *L. monocytogenes* challenge (Figure 1B). These results indicate that *L. monocytogenes* infection induced miR-146a expression in murine macrophages.

To further determine the expression pattern of miR-146a after *L. monocytogenes* infection in vivo, we challenged C57BL/6 mice with *L. monocytogenes* 10403S intraperitoneally for three days, and tested miR-146a expression in spleen and liver with real-time PCR. MiR-146a was up-regulated in both the spleen and liver (Figure 1C) after *L. monocytogenes* infection, while no significant increase was detected in either kidney or lung (Figure 1C). These results suggest that miR-146a may play an important role in macrophage-mediated defense against *L. monocytogenes*.

### 2.2. MiR-146a Knockout (KO) Mice Are Resistant to L. monocytogenes Infection

**Figure 2:**
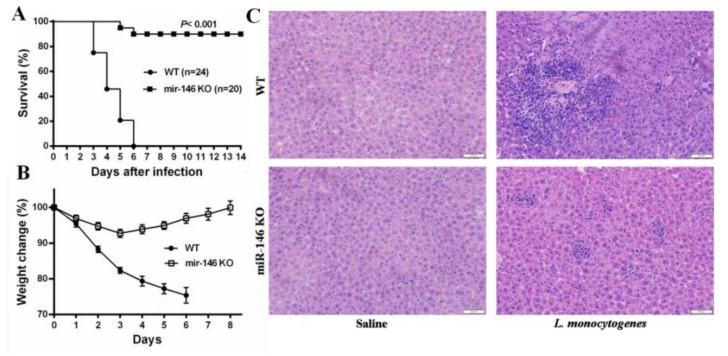
MiR-146a KO mice are resistant to *L. monocytogenes* infection. (**A**) Wild-type (WT) and miR-146a KO mice were infected i.p. with *L. monocytogenes* and survival was monitored daily for 14 days; (**B**) proportion of weight loss; (**C**) liver sections from *L. monocytogenes*-infected mice were prepared and stained with H&E for histological analysis (magnification, 200×). Data are shown as the mean ± S.E.M. of three independent experiments.

To characterize the role of miR-146a during *L. monocytogenes* infection, wild-type (WT) and miR-146a knockout (miR-146a KO) mice were infected intraperitoneally (i.p.) with a lethal dose of 10^6^ CFU of *L. monocytogenes*. Whereas, all of WT mice succumbed to infection within 5–6 days, 90.00% of miR-146a KO mice survived the infection and were still alive at day 14 post-infection (Figure 2A). WT mice lost, on average, 24.61% of their initial body weight before succumbing to infection, whereas weight loss in miR-146a KO mice was initially limited (<8% by day 3), and body weight gradually returned to normal after day 4 (Figure 2B).

*L. monocytogenes* infection triggers histopathological lesions and the formation of inflammatory cell foci, the size of which correlates with disease severity [23]. To examine the extent of immune cell infiltration, hematoxylin and eosin (H&E)-stained liver sections of *L. monocytogenes-*infected WT and miR-146a KO mice were compared. In agreement with our results above, the inflammatory cell foci in miR-146a KO livers were significantly smaller than those found in WT mice at day 3 post-infection (Figure 2C). These results indicate that miR-146a plays an important role in host defense against *L. monocytogenes* infection.

### 2.3. MiR-146a Deficiency Promotes Bacterial Clearance

**Figure 3:**
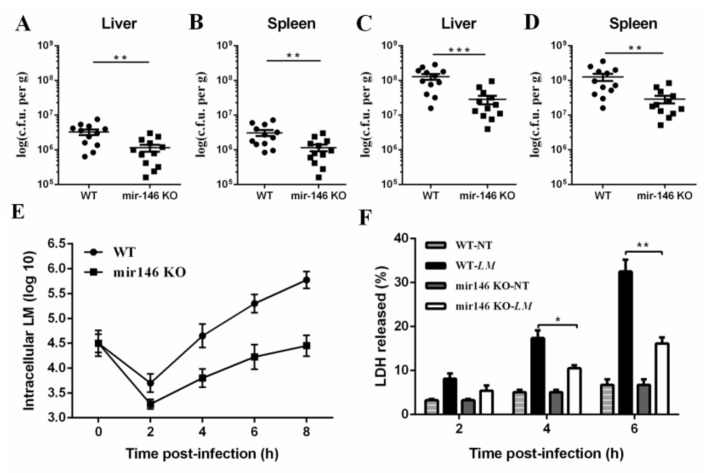
MiR-146a deficiency promotes bacterial clearance both in vitro and in vivo. (**A**) WT and miR-146a KO mice were infected with *L. monocytogenes* 10403S at a dose of 1 × 10^6^ CFU (i.p.). Mice were sacrificed on day 1 post-infection, and the bacterial load in the liver was determined (*n* = 12); (**B**) bacterial load in the spleen on day 1 (*n* = 12); (**C**) bacterial load in the liver on day 3 (*n* = 12); (**D**) bacterial load in the spleen on day 3 (*n* = 12); (**E**) WT and miR-146a KO BMDMs were incubated with *L. monocytogenes* 10403S at an MOI of 10 for 6 h, and the number of live bacteria in macrophages was determined; (**F**) lactate dehydrogenase (LDH) release in WT and miR-146a KO BMDMs. A total of 5 × 10^5^ BMDMs were infected for 6 h at an MOI of 10. Data are shown as the mean ± s.e.m. of three independent experiments. * *p*  <  0.05; ** *p*  <  0.01; *** *p*  <  0.001.

To examine whether the differential mortality between WT and miR-146a KO mice was associated with differences in bacterial clearance, we infected mice with *L. monocytogenes* at a dose of 1 × 10^6^ CFU bacteria administered intraperitoneally (i.p). Bacterial burdens in systemic organs were determined at days 1 and 3 post-infection. Notably, both the liver and spleen of miR-146a KO mice contained significantly fewer bacteria than those of WT mice at day 1 (Figure 3A,B), and this difference further increased by day 3 post-infection (Figure 3C,D). These results indicate that miR-146a plays an essential role in enhancing bacterial colonization in vivo.

To confirm whether the reduced bacterial burden in miR-146a KO mice correlated with the promoted killing of *L. monocytogenes* by macrophages, BMDMs of WT, and miR-146a KO were used to characterize bacterial clearance following *L. monocytogenes* challenge. BMDMs were incubated with *L. monocytogenes* in vitro at an MOI of 10. Intracellular bacteria were enumerated at several time points (Figure 3E). Induced bacterial growth was observed in BMDMs of miR-146a KO (Figure 3E), suggesting that miR-146a deficiency promoted the bacterial clearance of macrophages.

To examine whether the differential bacterial clearance in WT and miR-146a KO macrophages was associated with cell death, we assayed WT and miR-146a KO BMDMs infected with *L. monocytogenes* for release of lactate dehydrogenase (LDH). MiR-146a KO BMDMs showed significantly less LDH release than WT BMDMs at 4 h post-infection (Figure 3F), and this difference further increased at 6 h post-infection (Figure 3F). These results indicate that miR-146a deficiency promotes bacterial clearance both in vitro and in vivo.

### 2.4. MiR-146a Deficiency Alters the Gut Microbiota in Mice

The gut microbiota plays a critical role in bacterial infection [24]. To analyze the differences in gut microbiota between miR-146a KO than WT mice, we performed 16S rRNA V4 region sequencing analysis of the gut microbiota derived from stool samples of separately housed WT (*n* = 8) and miR-146a KO (*n* = 8) mice.

A total of 923,719 clean tags were obtained from the 16 samples by Illumina HiSeq 2500 sequencing. All of the sequences were delineated into operational taxonomic units (OTUs) with a 97% sequence similarity threshold. A total of 541 OTUs were obtained, and each sample contained 352–412 OTUs (Table S1). The richness estimators (observed-species and ACE) in the miR-146a KO group were significantly higher than in the WT group (*p* < 0.05) (Table S1). These results indicate that miR-146a deficiency could affect the abundance of gut microbiota. The increased abundance of gut microbiota helps to maintain health and to treat disease [25].

**Figure 4:**
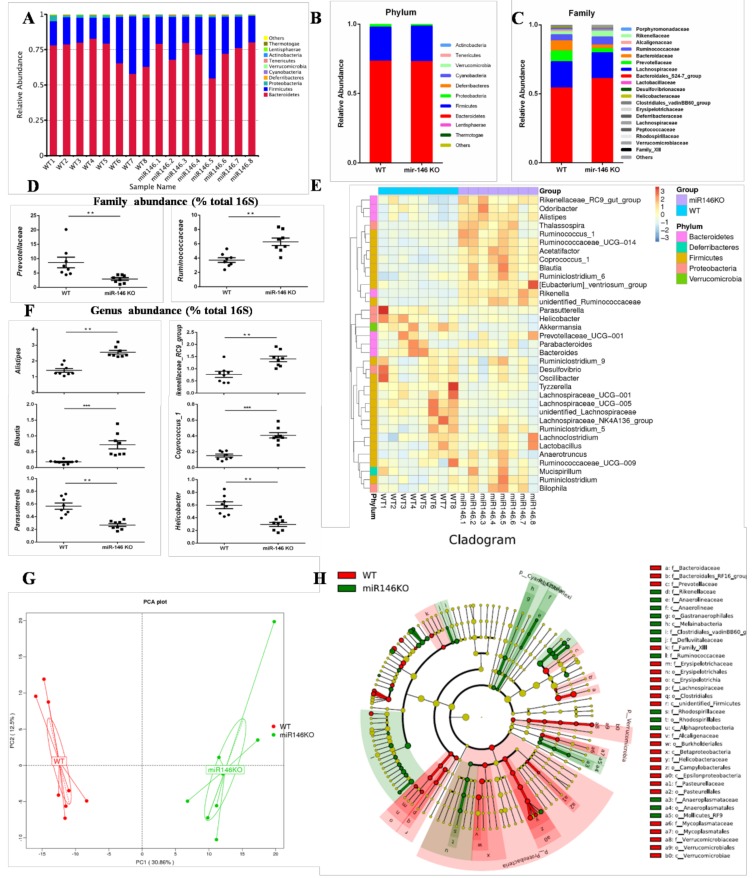
MiR-146a alters the gut microbiota in mice. (A) Bacterial-taxon-based analysis at the phylum level in the stool samples of WT (*n* = 8) and miR-146a KO (*n* = 8) mice; (**B**,**C**) gut microbiota structures are shown in histograms at the phylum and family levels in WT and miR-146a KO mice; (**D**,**F**) richness represented as the proportions of operational taxonomic units (OTUs) classified at the family and genus rank; (**E**) heatmap of relative abundances of bacterial taxa at the genus level; (**G**) principal component analysis (PCA) plot based on bacterial 16S rRNA gene sequence abundance in fecal content. Axes correspond to principal components 1 (*x* axis) and 2 (*y* axis); (**H**) analysis of gut microbiota at different taxonomic levels and key microbiota that contribute to the structure of gut microbiota in WT (in red) and miR-146a KO mice (in blue).

We then compared the gut microbiota composition in the WT and miR-146a KO mice. The gut microbiota was analyzed at the phylum, family, and genus levels. In total, all of the bacteria that were identified were classified into 176 genera, 60 families, and 15 phyla. At the phylum level, among the top 10 most abundant genera, *Bacteroidetes* and *Firmicutes* were particularly abundant in gut microbiota, not only at WT mice, but also at miR-146a KO mice (Figure 4A). *Bacteroidetes* were over 60% of the bacteria, and *Firmicutes* were approximately 20% (Figure 4B). The average proportion of *Firmicutes* (24.42% vs. 25.76%, *p* > 0.05), *Deferribacteres* (0.11% vs. 0.05%, *p* > 0.05), and *Cyanobacteria* (0.088% vs. 0.036%, *p* = 0.0019) were increased in miR-146a KO mice, while the abundances of *Proteobacteria* (1.10% vs. 1.95%, *p* = 0.002) and *Lentisphaerae* (0.0007% vs. 0.0054%, *p* = 0.037) were decreased (Figure 4B). In the miR-146a KO group, at the family level, *Prevotellaceae* (2.92% vs. 7.82%, *p* = 0.009) decreased significantly, whereas *Bacteroidales_S24-7_group* (60.95% vs. 54.20%, *p* > 0.05) and *Ruminococcaceae* (5.78% vs. 4.36%, *p* = 0.046) increased (Figure 4C,D). At the genus level, *Alistipes* (2.36% vs. 1.40%, *p* = 0.002), *Rikenellaceae_RC9_group* (1.41% vs. 0.77%, *p* = 0.003), *Blautia* (0.72% vs. 0.17%, *p* = 0.001), *unidentified Ruminococcaceae* (0.84% vs. 0.26%, *p* = 0.001), *Ruminiclostridium* (0.40% vs. 0.28%, *p* = 0.04), *Coprococcus_1* (0.39% vs. 0.15%, *p* = 0.001), *Ruminococcaceae_UCG-014* (0.41% vs. 0.11%, *p* = 0.001), and *Ruminococcus_1* (0.091% vs. 0.036%, *p* = 0.01) increased significantly in the miR-146a KO group, while *Parasutterella* (0.29% vs. 0.64%, *p* = 0.005), *Desulfovibrio* (0.28% vs. 0.49%, *p* = 0.03), and *Helicobacter* (0.29% vs. 0.59%, *p* = 0.01) decreased (Figure 4E,F).

The gut microbiota was compared using multivariate principal component analysis (PCA). Sample plots in the PCA indicated different trends in WT and miR-146a KO mice (Figure 4G). We compared the gut microbiota in WT and miR-146a KO mice using LEfSe to identify the specific bacterial taxa. A cladogram representing the structure of the gut microbiota is shown in Figure 4H. The greatest differences in the multiple levels of taxa between the two communities are displayed (Figure 4H). These data indicate that the significantly increased *Proteobacteria* (phylum) could be one of the biomarkers of WT mice. *Ruminococcaceae* (family) could be a biomarker of the miR-146a KO group.

Overall, our 16S rRNA gene sequencing analyses revealed a markedly distinct intestinal microbial landscape between WT and miR-146a KO mice. Furthermore, miR-146a deficiency in mice contributed to maintaining a healthy composition of the gut microbiota. These results suggest that miR-146a deficiency altered the gut microbiota in mice. As far as we know, this is the first direct evidence that miR-146a shaped the gut microbiota in mice.

### 2.5. MiR-146a Deficiency Protects against L. monocytogenes Infection by Modulating the Gut Microbiota

**Figure 5:**
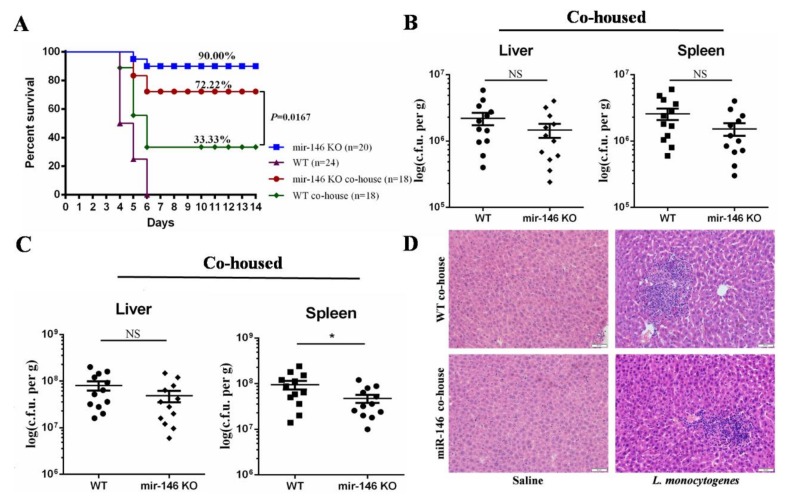
MiR-146a deficiency protects against *L. monocytogenes* infection *by regulating* the gut microbiota. (**A**) miR-146a KO co-housed with WT (miR-146a co-house) and WT co-housed with miR-146a KO mice (WT co-house) were infected i.p. with *L. monocytogenes* and survival was monitored daily for 14 days; (**B**) bacterial loads in the liver and spleen on day 1 (*n* = 12); (**C**) bacterial loads in the liver and spleen on day 3 (*n* = 12); (**D**) liver sections from *L. monocytogenes*-infected mice were prepared and stained with H&E for histological analysis (magnification, 200×). Each point represents an individual mouse and the mean ± s.e.m; *p* values were determined by the unpaired two-tailed test. The results show cumulative data from two different experiments. * *p*  <  0.05; NS, no significance.

Because miR-146a KO mice had some alterations in gut microbiota composition as compared to WT controls, we took advantage of the transmissible nature of the gut microbiota to investigate whether the susceptibility to *L. monocytogenes* infection could be increased in miR-146a KO mice by co-housing them with WT mice. After four weeks of co-housing, we analyzed the co-housing gut microbiota by qPCR. Our 16S rRNA gene sequencing analyses revealed that there was a significantly difference in gut microbiota between separately housed WT and miR-146a KO mice, including *Proteobacteria*, *Prevotellaceae*, and *Alistipes* (Figure 4B,D,F). Interestingly, co-housing WT and miR-146a KO mice equilibrated the relative abundance of *Proteobacteria, Prevotellaceae*, and *Alistipes* in WT and miR-146a KO mice (Figure S1). These results indicated that co-housing could promote the exchange of gut microbiota between WT and miR-146a KO mice. The co-housed mice were infected intraperitoneally (i.p.) with a lethal dose of 10^6^ CFU of *L. monocytogenes*. We found that WT mice co-housed with miR-146a KO mice (33.33.00%, 6/18) significantly increased the survival when compared with separately housed WT mice (0.00%, 0/24) (Figure 5A), while the survival rate of co-housed miR-146a KO mice (72.22%, 13/18) slightly decreased in comparison with separately housed miR-146a KO mice (90.00%, 18/20) (Figure 5A).

Bacterial counts in the liver and spleen of co-housed mice were not significantly different at day 1 post-infection (Figure 5B). Moreover, the difference of bacterial burden between co-housed WT and miR-146a KO mice decreased at day 3 post-infection, as compared with that of separately housed mice (Figure 3D and Figure 5C). Inflammatory cell foci in co-housed miR-146a KO mouse livers were slightly smaller than those that werefound in co-housed WT mice at day 3 post-infection (Figure 5C). Collectively, this data suggest that the gut microbiota contributes to protection of miR-146a deficiency against *L. monocytogenes* infection.

## 3. Discussion

MiRNAs play essential roles in host defense against bacterial infection [4]. Several miRNAs are induced in macrophages during *L. monocytogenes* infection, including miR-155, miR-21, miR-146a, miR-125a-3p/5p, and miR-149 [26,27]. In the current study, it remains unclear whether *L. monocytogenes*-triggered miRNAs regulate bacterial clearance and the outcome of infections. Our results reveal a novel role of miR-146a in regulating *L. monocytogenes* infection. Our data show that miR-146a deficiency protects against L. monocytogenes infection. These findings provide a better understanding of the role of miR-146a in regulating host defense against *L. monocytogenes* infection.

MiR-146a plays critical roles in innate immunity, inflammation, antiviral response, and cancer [28]. MiR-146a is up-regulated after many microbial infections. Our results suggest that miR-146a is highly up-regulated in RAW264.7 cells and BMDMs in response to *L. monocytogenes* infection. Similarly, miR-146a is increased in the spleen and liver of mice that are infected with *L. monocytogenes*. MiR-146a plays an important role in the interaction between pathogen and host. A recent study showed that miR-146a inhibits iNOS expression and NO generation in macrophages by targeting TRAF6, and thus suppressing mycobacterial clearance [15]. In this work, we illustrate a new regulatory role of miR-146a during *L. monocytogenes* infection. We found that miR-146a KO mice were highly resistant to *L. monocytogenes* infection. This was manifested by a longer survival time and a higher survival rate. After *L. monocytogenes* infection, miR-146a KO mice were more efficient in controlling bacterial replication, and they exhibited less pathological damage. Furthermore, miR-146a deficiency in BMDMs resulted in reduced cell death and intracellular survival of *L. monocytogenes*. These data suggest that miR-146a deficiency protects against *L. monocytogenes* infection.

The gut microbiota plays a key role in host defense against bacterial infection. It not only provides direct colonization resistance against pathogens, but it is increasingly considered to be an important modulator of host defense [25]. Studies have shown that miRNAs play pivotal roles in the interaction between host and gut microbiota [29], but it is not known whether miR-146a can regulate gut microbiota. Thus, we compared the gut microbiota of wild-type and miR-146a KO mice by high-throughput sequencing of 16S rRNA. We found that there were significant differences in gut microbiota between miR-146a KO and WT mice. When compared with WT mice, miR-146a deficiency led to an increase in the abundance of gut microbiota. Reduced community richness of the gut can increase the attachment and invasion of pathogen [30]. At the phylum level, we observed a reduction in *Proteobacteria* in miR-146a KO mice. Data show that *Proteobacteria* may be a potential microbial signature of disease [31]. The decrease in *Proteobacteria* helps to ensure the stability of gut microbiota in miR-146a KO mice. In addition, our results suggest that the number of the family *Prevotellaceae* was significantly decreased compared to WT mice. Gut microbiota dysbiosis contributes to the development of inflammatory bowel diseases (IBD), and *Prevotellaceae* can exacerbate colitis or drive chronic intestinal inflammation [32]. When compared to WT mice, miR-146a KO mice had increased levels of *Alistipes*, *Blautia*,* Coprococcus_1*, and *Ruminococcus_1*, and decreased levels of *Parasutterella*. The major metabolite of *Alistipes* is succinic acid. Succinic acid can enhance the gut barrier function by facilitating tight junction assembly [33]. Interestingly, these bacteria all belong to the SCFA-producing bacteria, including *Blautia*,* Coprococcus_1* and *Ruminococcus_1*. For example, *Blautia* mainly produces acetic acid [34], while *Coprococcus_1* and *Ruminococcus_1* produce butyrate [35]. Accumulating evidence suggests that short-chain fatty acids (SCFA) help to improve intestinal barrier function, and play a protective role in the host defense against intestinal diseases [36]. In Crohn’s disease patients, the gut microbiota shows an increase the number of Parasutterella as compared with healthy controls [37]. The decrease in *Parasutterella* is beneficial to maintain intestinal homeostasis. These results indicated that the abundance and composition of gut microbiota in miR-146a KO mice was significantly changed compared with WT mice. Furthermore, miR-146a-deficient in mice contributed to maintaining a healthy composition of the gut microbiota. MiR-146a plays an important role in health and disease, but it is not known whether miR-146a can regulate gut microbiota. Overall, our findings provide the first direct evidence that miR-146a can shape the gut microbiota.

The gut microbiota plays an important role in host defense against *L. monocytogenes* infections [24]. We found that miR-146a KO mice enhanced resistance to L. monocytogenes infection. The gut microbiota can be counterbalanced by co-housing [38]. To determine whether miR-146a-mediated alterations of gut microbiota are associated with *L. monocytogenes* infections, we co-housed WT with miR-146a KO mice. Interestingly, WT co-housed with miR-146a KO mice for four weeks had increased resistance to L. monocytogenes infection. Co-housed miR-146a KO mice were still more resistant to L. monocytogenes infection when compared with co-housed WT mice. These results indicate that miR-146a-mediated alterations of gut microbiota can regulate the outcome of infection with L. monocytogenes.

In summary, the results of this study reveal a novel role of miR-146a during *L. monocytogenes* infection. MiR-146a deficiency enhanced resistance to *L. monocytogenes* infection. By high-throughput sequencing 16S rRNA, our results provide the first direct evidence that miR-146a can shape the gut microbiota in mice. MiR-146a KO mice contributed to maintaining a healthy composition of the gut microbiota. Moreover, miR-146a deficiency guides the gut microbiota to alleviate infection. These results demonstrate that miR-146a deficiency protects against *L. monocytogenes* infection by regulating the gut microbiota. Further studies are needed to elucidate the relationships between miR-146a and the gut microbiota. In addition, miR-146a may provide new therapeutic targets for the treatment of *L. monocytogenes* infection.

## 4. Material and Methods

### 4.1. Mice

MicroRNA-146a knockout (miR-146a KO) and wild-type (WT) C57BL/6J mice were purchased from The Jackson Laboratory (Bar Harbor, ME, USA). All mice were 6–8 weeks old at the time of experimentation. The mice were maintained on a 12-h dark-light cycle and allowed for free access to food and tap water under controlled temperatures. For co-housing experiments, equal numbers of female WT and miR-146a KO mice were housed in the same cage for four weeks [38]. All of the animal studies were conducted according to the Institutional Animal Care and Use Committee of Jilin University (approved on 30 September 2016, Protocol No. 20160930).

### 4.2. Cell Culture

RAW264.7 cells were cultured in Dulbecco’s modified Eagle’s medium (DMEM) (Gibco, Thermo Fisher Scientific, Waltham, MA, USA) without antibiotics at 37 °C under a 5% CO_2_ atmosphere. BMDMs were prepared and cultured as previously described [39]. Briefly, BMDMs were isolated from the femurs and tibias of 8- to 10-week-old mice and were cultured in RPMI-1640 medium containing 10% heat-inactivated FBS and 25% L929 conditioned medium. The cells were harvested for assays after seven days of differentiation.

### 4.3. L. monocytogenes Infection In Vivo

*L. monocytogenes* 10403S was a gift from Wei-Huan Fang (Microbiology Institute of Preventive Veterinary Medicine, Zhejiang University, Hangzhou, China). Lethal *L. monocytogenes* infection was established by infecting 6–8-week-old female mice with 1 × 10^6^ CFU bacteria administered intraperitoneally (i.p.). Animals were weighed and monitored daily for mortality for up to 14 days. Differences in survival were analyzed by Mantel-Cox test. To study bacterial clearance, mice were infected with *L. monocytogenes* at a dose of 1 × 10^6^ CFU (i.p.). Mice were sacrificed at days 1 and 3 post-infection, liver and spleen tissue were homogenized, and bacterial burdens were enumerated by serial dilution on Brain-Heart-Infusion agar or plates, as described previously [40]. Mice were sacrificed at day 3 post-infection, and different organs (liver, spleen, kidney, and lung) were collected for RNA isolation.

### 4.4. L. monocytogenes Infection In Vitro

RAW264.7 cells and BMDMs were seeded in 6-well cell culture plates and cultured overnight in antibiotic-free medium. After treatment with *L. monocytogenes* 10403S (MOI = 20) for 30 min, the cells were washed in prewarmed medium and supplemented with RPMI-1640 medium containing a cocktail of antibiotics (200 U/mL penicillin, 200 U/mL streptomycin, and 100 μg/mL gentamicin) for an additional 3 or 6 h. Cells were collected for RNA isolation.

BMDMs were infected with *L. monocytogenes* 10403S at an MOI of 10 at 37 °C. After incubation for 30 min, the extracellular bacteria were eliminated with a cocktail of antibiotics (200 U/mL penicillin, 200 U/mL streptomycin, and 100 μg/mL gentamicin) for 8 h. After treatment, the cells were lysed using 0.1% Triton X-100 (Sigma Chemical Co, St Louis, MO, USA). Bacterial counts were obtained by plating serial dilutions of cell lysate on BHI agar plates. Data are shown as the mean CFU/mL ± structural equation modeling (SEM).

### 4.5. RNA Isolation and Real-Time PCR

Total RNA was extracted from cells and tissue samples using the TRIzol^®^ Reagent (Invitrogen, Carlsbad, CA, USA) according to the manufacturer’s protocol. MiR-146a was quantified by RT-PCR using specific Taqman assays for miRNA (Applied Biosystems, Foster City, CA, USA) and specific primers for miR-146a (Applied Biosystems, primer identification numbers: 000468 for mmu-miR-146a and 001973 for snRU6). MiR-146a was quantified with the 2^−ΔΔ*C*t^ relative quantification method with normalization to the U6 small nucleolar RNA (snRU6).

### 4.6. Cell Death Assay

Cell death was evaluated by detection of the cytoplasmic enzyme LDH. The LDH assay was performed by using a Promega cytotoxicity kit (Promega, Madison, WI, USA), according to the manufacturer’s instructions.

### 4.7. Histopathology

Formalin-preserved liver and spleen sections were processed and embedded in paraffin by standard techniques. Longitudinal sections that were 5 μm thick were stained with hematoxylin and eosin (H&E) and were examined by a pathologist that was blinded to the experimental groups.

### 4.8. Sample Collection

Mouse feces were sampled from WT mice (*n* = 8) and miR-146a KO mice (*n* = 8). All of the mice were transferred to fresh sterilized cages and the feces were collected from the cages within two hours. All of the samples were immediately frozen and stored at −80 °C until further treatment.

### 4.9. High-Throughput Sequencing of 16S rRNA

DNA from the stool samples was pooled and purified using the QIAamp DNA Stool Mini Kit (Qiagen, Valencia, CA, USA) according to the manufacturer’s instructions. Quantification was performed with a NanoDrop ND-2000 UV-Vis spectrophotometer (NanoDrop Technologies, Wilmington, DE, USA). Sixteen libraries were constructed and sequenced using the Illumina MiSeq sequencing platform. The V4 regions of the 16S rRNA gene comprising were amplified by PCR using specific bacterial primers (Table S2). High-throughput sequencing was performed using the Illumina MiSeq platform, following the manufacturer’s instructions at Novogene Bioinformatics Technology Co., Ltd. (Beijing, China). Sequences were processed using the QIIMEpipeline (version 1.8.0, available online: http://qiime.org). The quality-filtered reads were clustered into operational taxonomic units (OTUs) at a 97% identity level. Taxonomies were assigned with the UCLUST algorithm against the GreenGenes database (gg_otus_13_8). Alpha diversity was calculated using the observed species metrics. To calculate the beta diversity (weighted UniFrac distance), 8000 sequences were randomly selected from each sample. Statistical significance of the factors that were potentially contributing to compositional differences between the samples was examined using Adonis, ANOSIM, and MRPP, which were performed using QIIME 1.8.0. LEfSe was used for detecting the differences in the abundance of bacterial species between groups.

The raw reads were deposited into the NCBI Sequence Read Archive (SRA) database (accession number: SRP093952).

### 4.10. Gut Microbiota Analysis by qPCR

DNA was isolated from stools (200 mg/mouse) from each mouse using TIANGEN DNA Stool Mini Kit. The abundance of bacterial populations in mouse stools was measured by qPCR using the ABI 7500 Sequence Detection System. The PCR mix was made with SYBR Green Master Mix (Roche, Mannheim, Germany). The thermo cycling program was 3 min at 95 °C and 40 cycles of 15 sec at 95 °C and 1 min at 60 °C. The following specific forward and reverse primers, respectively, were used: *Proteobacteria* (5′-TCGTCAGCTCGTGTYGTGA-3′ and 5′-CGTAAGGGCCATGATG-3′) [41], *Prevotellaceae* (5′-CGGAAGGTCCGGGCGTTATCCG-3′ and 5′-CCTGTTCGATACCCGCGCCTTC-3′) [42], *Alistipes* (5′-GTACTAATTCCCCATAACATTCGAG-3′ and 5′-CTAATACAACGCATGCCCATCTT-3′) [43], and the universal 16S rRNA gene EUB primers (5′-AGAGTTTGATCCTGGCTC-3′ and 5′-TGCTGCCTCCCGTAGGAGT-3′) [42]. The relative abundance of each bacterial population was quantified with the 2^−ΔΔ*C*t^ relative quantification method with normalization to the universal 16S rRNA gene EUB.

### 4.11. Statistical Analysis

All of the results were obtained from three independent experiments. The results are shown as the means ± SEM. Differences between mean values were determined with student’s *t*-test. *p* < 0.05 was considered statistically significant. Statistics were performed with Prism software (GraphPad, La Jolla, CA, USA).

## Supplementary Materials

The following are available online at http://www.mdpi.com/1422-0067/19/4/993/s1.
