# Supplementary material for: MicroRNA-146a Deficiency Protects against Listeria monocytogenes Infection by Modulating the Gut Microbiota
Source: Int J Mol Sci. 2018 Mar 26;19(4):993. doi: 10.3390/ijms19040993 (PMC5979314; doi:10.3390/ijms19040993)
Supplement: Supplementary file 1 [file ijms-19-00993-s001.pdf]

Supplementary Materials

Table S1. Analysis of 16S rRNA gene sequences.

Sample Name	No.reads	Observed -species	Shannon	Simpson	Chao1	ACE	Good's coverage
WT 1	59,243	353	5.933	0.961	419.300	387.581	0.998
WT 2	50,362	355	6.218	0.972	366.447	372.208	0.999
WT 3	59,288	362	5.860	0.957	417.000	403.746	0.998
WT 4	67,117	360	5.905	0.964	465.286	412.770	0.998
WT 5	62,160	352	5.917	0.964	422.714	394.597	0.998
WT 6	48,838	353	6.137	0.967	373.517	375.591	0.999
WT 7	63,900	370	5.969	0.959	403.176	403.059	0.999
WT 8	61,243	362	6.189	0.969	397.037	392.522	0.999
miR146a KO 1	55,575	383	6.093	0.961	414.364	409.697	0.999
miR-146a KO 2	52,511	394	6.248	0.964	404.568	408.526	0.999
miR-146a KO 3	63,629	412	6.147	0.965	455.872	448.665	0.998
miR-146a KO 4	64,696	392	6.386	0.97	420.447	420.744	0.999
miR-146a KO 5	67,937	410	6.552	0.975	455.370	440.006	0.999
miR-146a KO 6	60,355	405	6.356	0.971	433.286	429.992	0.999
miR-146a KO 7	50,537	374	6.055	0.965	398.118	398.281	0.999
miR-146a KO 8	56,328	370	5.819	0.953	416.406	406.935	0.998

Number of sequences, observed-species, Shannon index, simpson index, Chao1 index, ACE index and Good's coverage calculated for the microbial communities of lamb packaged in the VP and MAP environments for each storage period for high-throughput sequence reads determined at a 97% similarity.

Table S2. Sequences of 16S rRNA V4 primer.

16S rRNA V4 primers	Sequence (5′ to 3′)
515F	GTGCCAGCMGCCGCGGTAA
806R	GGACTACH-VGGGTWTCTAAT

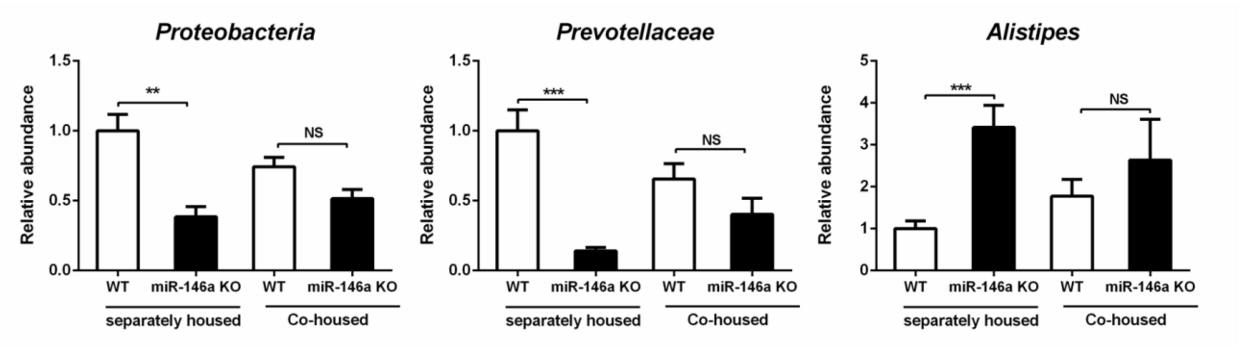

Figure S1. Relative abundance of bacterial populations in separately housed and co-housed mice. The results are means ± SEM of 8 mice/group; significance of differences: *P < 0.05; **P < 0.01; ***P < 0.001; NS, no significance.
